# Global marine redox changes drove the rise and fall of the Ediacara biota

**DOI:** 10.1111/gbi.12359

**Published:** 2019-07-28

**Authors:** Feifei Zhang, Shuhai Xiao, Stephen J. Romaniello, Dalton Hardisty, Chao Li, Victor Melezhik, Boris Pokrovsky, Meng Cheng, Wei Shi, Timothy M. Lenton, Ariel D. Anbar

**Affiliations:** ^1^ School of Earth Sciences and Engineering Nanjing University Nanjing China; ^2^ Department of Geology and Geophysics Yale University New Haven CT USA; ^3^ The Globe Institute University of Copenhagen Copenhagen K Denmark; ^4^ School of Earth and Space Exploration Arizona State University Tempe AZ USA; ^5^ Department of Geosciences Virginia Tech Blacksburg VA USA; ^6^ Department of Earth and Environmental Science Michigan State University East Lansing MI USA; ^7^ State Key Laboratory of Biogeology and Environmental Geology China University of Geosciences Wuhan China; ^8^ Geological Survey of Norway Trondheim Norway; ^9^ Geological Institute Russian Academy of Sciences Moscow Russia; ^10^ Global Systems Institute University of Exeter Exeter UK; ^11^ School of Molecular Science Arizona State University Tempe AZ USA

**Keywords:** early animals, Neoproterozoic, ocean oxygenation, Shuram negative carbon isotope excursion, uranium isotopes

## Abstract

The role of O_2_ in the evolution of early animals, as represented by some members of the Ediacara biota, has been heavily debated because current geochemical evidence paints a conflicting picture regarding global marine O_2_ levels during key intervals of the rise and fall of the Ediacara biota. Fossil evidence indicates that the diversification the Ediacara biota occurred during or shortly after the Ediacaran Shuram negative C‐isotope Excursion (SE), which is often interpreted to reflect ocean oxygenation. However, there is conflicting evidence regarding ocean oxygen levels during the SE and the middle Ediacaran Period. To help resolve this debate, we examined U isotope variations (δ^238^U) in three carbonate sections from South China, Siberia, and USA that record the SE. The δ^238^U data from all three sections are in excellent agreement and reveal the largest positive shift in δ^238^U ever reported in the geologic record (from ~ −0.74‰ to ~ −0.26‰). Quantitative modeling of these data suggests that the global ocean switched from a largely anoxic state (26%–100% of the seafloor overlain by anoxic waters) to near‐modern levels of ocean oxygenation during the SE. This episode of ocean oxygenation is broadly coincident with the rise of the Ediacara biota. Following this initial radiation, the Ediacara biota persisted until the terminal Ediacaran period, when recently published U isotope data indicate a return to more widespread ocean anoxia. Taken together, it appears that global marine redox changes drove the rise and fall of the Ediacara biota.

## 1 INTRODUCTION

After life first emerged more than three billion years ago, single‐celled organisms dominated the planet for most of its history. It is not until the Ediacaran Period (635–541 Ma) that large and morphologically complex multicellular eukaryotes became abundant and diverse (Yuan, Chen, Xiao, Zhou, & Hua, 2011). The Ediacara biota, which characterizes the second half of the Ediacaran Period, arose in the middle Ediacaran Period (Xiao & Laflamme, 2009), reached their maximum taxonomic diversity and morphological disparity about 560 Ma, and then declined in the terminal Ediacaran Period (~550–541 Ma) (Darroch, Smith, Laflamme, & Erwin, 2018; Laflamme, Darroch, Tweedt, Peterson, & Erwin, 2013; Shen, Dong, Xiao, & Kowalewski, 2008; Xiao & Laflamme, 2009). Although the phylogenetic affinities of members of the Ediacara biota remain controversial, it is clear that some of them represent mobile macrometazoans, including putative cnidarian‐grade animals (Liu, McLlroy, & Brasier, 2010) and bilaterians (Gehling, Runnegar, & Droser, 2015). Importantly, most taxa of the Ediacara biota, and certainly the White Sea and Nama assemblages, appear to be bracketed by two negative carbon isotope excursions (Darroch et al., 2018), raising the intriguing possibility that the rise and fall the Ediacara biota may have been related to environmental and ecological events.

Recent studies provide evidence that an episode of extensive marine anoxia during the terminal Ediacaran Period may have contributed to the decline of the Ediacara biota (Tostevin et al., 2019; Wei et al., 2018; Zhang, Xiao, et al., 2018). However, the cause of the rise of the Ediacara biota during the middle Ediacaran Period remains a subject of intensive debate. A temporal correlation with evidence for a major redox transition suggests that a profound ocean oxygenation event may have sparked this evolutionary event (Canfield, Poulton, & Narbonne, 2007; Fike, Grotzinger, Pratt, & Summons, 2006; McFadden et al., 2008; Shi et al., 2018). However, others have argued that the diversification of bilaterians may have been enabled by the evolution of key developmental toolkits (Erwin, 2009) and/or that the rise of eumetazoans was driven by positive ecological feedbacks (Butterfield, 2007).

The oxygenation hypothesis is attractive because aerobic metabolic pathways provide much more energy than anaerobic ones, and so the presence of free O_2_ is often regarded as a prerequisite for the evolution of macroscopic animals, particularly those involved in energetically expensive lifestyles such as mobility, burrowing, and predation (Sperling et al., 2013). Given the importance of O_2_ for animal physiology, researchers have combed Neoproterozoic successions to determine when there were significant changes in the proportion of oxic to anoxic waters in the deep ocean (Canfield et al., 2008, 2007; Fike et al., 2006; Johnston et al., 2013; McFadden et al., 2008; Sperling et al., 2015).

Carbonate sedimentary rocks from the middle Ediacaran Period have attracted special attention (Fike et al., 2006; Grotzinger, Fike, & Fischer, 2011; Li et al., 2017; McFadden et al., 2008), because they offer an opportunity to clarify the relationship between a redox event and the rise of the Ediacara biota. Middle Ediacaran carbonates in many parts of the world (including South China, Siberia, western United States, Oman, and South Australia) record the largest negative δ^13^C_carb_ excursion (<−12‰) in Earth history, termed the “Shuram excursion” (SE) after its initial discovery in the Shuram Formation of Oman (Burns & Matter, 1993; Grotzinger et al., 2011). When the Ediacara biota and the SE are recorded in the same succession, the former always postdate the latter (Xiao et al., 2016), with only one possible exception in the southeastern Mackenzie Mountains where rangeomorph, arboreomorph, and erniettomorph Ediacara fossils predate a negative δ^13^C_carb_ excursion (−2‰) interpreted as a putative equivalent of SE (Macdonald et al., 2013; Narbonne et al., 2014). Thus, the rise of the Ediacara biota, particularly the appearance of large, mobile, and morphologically complex animals, may have occurred either during (Darroch et al., 2018) or immediately following the SE (e.g., McFadden et al., 2008; Xiao et al., 2016). As such, it has been proposed that the SE represents an unprecedented ocean oxygenation event, which sparked the diversification of complex animals (Fike et al., 2006; McFadden et al., 2008; Shi et al., 2018; Wood et al., 2015). However, the extent of global ocean redox change across this critical interval is poorly constrained (Bristow and Kennedy, 2008). For instance, proxies for tracking local or regional Fe‐S‐C systematics and iodine chemistry have been used to infer oxygenation of the deep ocean in some locations during or after the SE (Fike et al., 2006; Hardisty et al., 2017; McFadden et al., 2008; Wood et al., 2015). However, similar data from other localities have been used to argue for a persistence of redox‐stratified and ferruginous marine environments during this critical interval (Canfield et al., 2008; Johnston et al., 2013; Li et al., 2010; Sahoo et al., 2016; Sperling et al., 2015). These contrasting views likely arise because these paleoredox proxies are inherently local or indirect tracers of oxygenation.

The U isotope system (^238^U/^235^U, denoted as δ^238^U) measured in carbonate sedimentary rocks is a more direct probe of global ocean redox conditions and can be used to place quantitative constraints on the extent of global redox changes (Brennecka, Herrmann, Algeo, & Anbar, 2011; Clarkson et al., 2018; Elrick et al., 2017; Lau et al., 2016; Tostevin et al., 2019; Wei et al., 2018; Zhang, Algeo, Cui, et al., 2019; Zhang, Algeo, Romaniello, et al., 2018; Zhang, Romaniello, et al., 2018; Zhang, Xiao, et al., 2018). The power of U isotopes as a global proxy derives from the fact that in the modern ocean U has a long residence time, ~500 kyr (Dunk, Mills, & Jenkins, 2002), and hence, δ^238^U is uniform in the open ocean (e.g., Tissot & Dauphas, 2015). Although the concentration and residence time of U in seawater would both be reduced during times of expanded marine anoxia, studies suggest that the U isotope composition of open ocean seawater was likely uniform even during periods of expanded anoxia (Clarkson et al., 2018; Zhang, Algeo, Romaniello, et al., 2018; Zhang, Xiao, et al., 2018). Seawater δ^238^U varies with redox conditions because the reduction of dissolved U(VI) to U(IV), which is immobilized in anoxic sediments, results in a large and detectable change in δ^238^U (0.6‰–0.85‰), favoring the ^238^U over ^235^U in the reduced species (Andersen et al., 2014). Thus, δ^238^U of U(VI) dissolved in seawater decreases as the global areal extent of bottom water anoxia increases (Brennecka et al., 2011). Marine carbonate sediments have been demonstrated to record the δ^238^U of seawater, subject to a 0.2‰–0.4‰ offset, which likely reflects incorporation of U(IV) into shallow sediments from anoxic porewaters (Chen et al., 2018; Romaniello, Herrmann, & Anbar, 2013; Tissot et al., 2018). Studies comparing the trends and absolute values of δ^238^U in coeval Permian–Triassic carbonate sediments from around the world have shown excellent agreement, demonstrating that carbonates may provide a robust record of variations in seawater δ^238^U (Brennecka et al., 2011; Elrick et al., 2017; Lau et al., 2016; Zhang, Algeo, Romaniello, et al., 2018; Zhang, Romaniello, et al., 2018).

**Figure 1:**
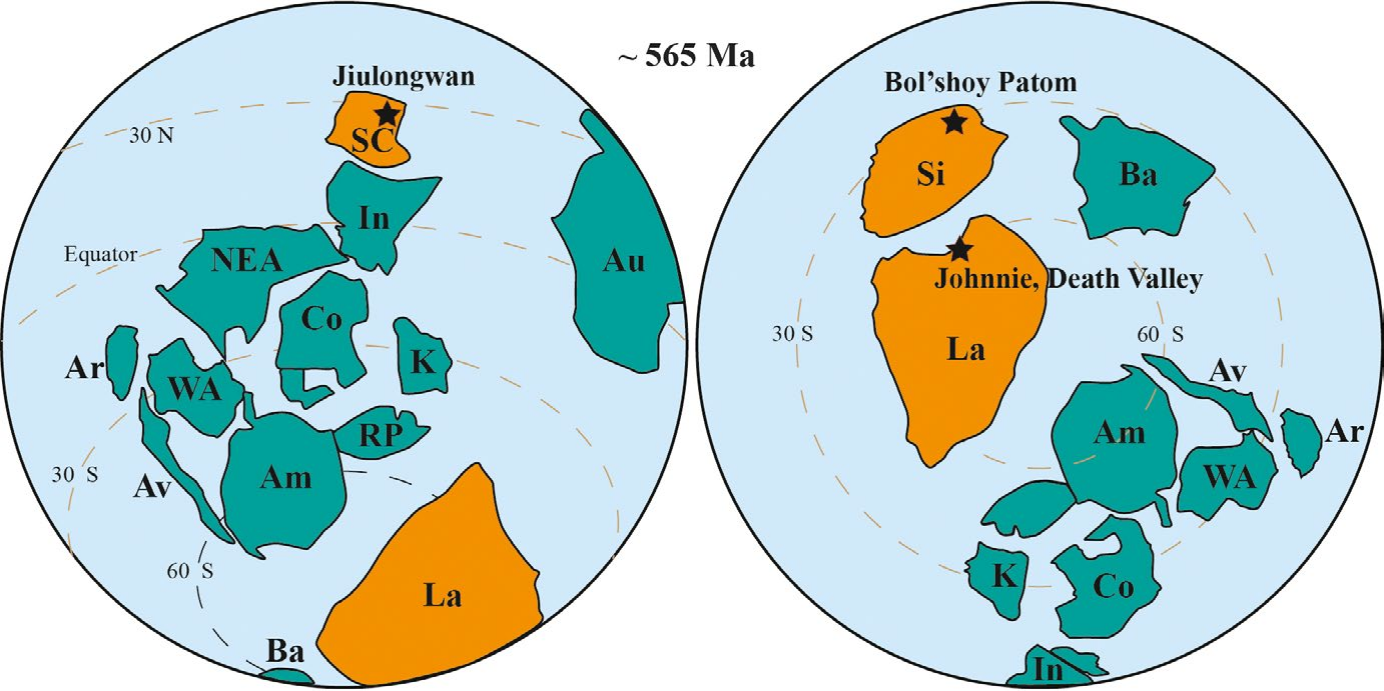
Paleogeography at ~565 Ma (modified after Meert & Lieberman, 2008)**.** The black stars in the maps show the locations of study sections. Am, Amazonia; Ar, Armorica; Au, Australia; Av, Avalonia; Ba, Baltica; Co, Congo; I, India; K, Kalahari; La, Laurentia; NEA, NE Africa; RP, Rio Plata; S, Sahara; SC, South China; Si, Siberia; WA, Western Africa [Colour figure can be viewed at http://wileyonlinelibrary.com]

To obtain new constraints on the extent of global redox change across the SE event, we applied the U isotope proxy and associated major and trace elements to carbonates across the SE from three widely separated sections: the Jiulongwan section in South China; the Bol'shoy Patom section in Siberia; and the Death Valley section (the Johnnie Formation) in the western United States (Figure 1).

## 2 STUDY SECTIONS

The SE at the Jiulongwan section (GPS: N 30°48'15.05″, W 111°3'18.61″) is represented by the Doushantuo Member III (Li et al., 2017; McFadden et al., 2008), which is about 70 m thick. The lower 40 m is composed of dolostone with bedded chert, and the upper 30 consists primarily of ribbon limestone (see figure 4 in the Supplementary Information of McFadden et al., 2008 for a detailed summary of the stratigraphy at the Jiulongwan section). Sedimentological evidence suggests that the entire Doushantuo Formation at Jiulongwan was deposited below or near the wave base (McFadden et al., 2008). Though there are some debates about the detailed depositional environments (Jiang, Shi, Zhang, Wang, & Xiao, 2011; McFadden et al., 2008; Zhu, Zhang, & Yang, 2007)—for example, some studies suggest deposition in a locally restricted setting (e.g., Jiang et al., 2011), the Doushantuo Formation at Jiulongwan was more likely to have been accumulated in a shelf basin that was connected to the open ocean (see McFadden et al., 2008 for details). Forty‐six samples from the Jiulongwan section were analyzed for U isotopes.

The SE at the Bol'shoy Patom section is represented by the Kholychskaya Formation, the Alyanchskaya Formation, and the Nikol'skaya Formation, which are ~200, ~530, and ~390 m thick, respectively, and are composed of well‐preserved high‐Sr limestone (Melezhik, Pokrovsky, Fallick, Kuznetsov, & Bujakaite, 2009). Sedimentary facies associations suggest deposition on a shallow carbonate platform that was well connected to the open ocean with neither basin isolation nor chemical or physical stratification (see Melezhik et al., 2009 for details). Forty‐five samples from the Bol'shoy Patom section were analyzed for U isotopes.

The SE in the Death Valley region, California, comes from Saddle Peak Hills (GPS: N 35°45.439′, W 116°20.936′) and is represented by the Rainstorm Member of the Johnnie Formation, which is >100 m thick in the study section and is composed of interbedded siltstone, sandstone, and conglomerate, with locally abundant dolostone. Sedimentary features suggest deposition in distal‐fluvial and shallow‐marine (above storm wave base) conditions (Verdel, Wernicke, & Bowring, 2011). The Shuram δ^13^C_carb_ excursion occurs primarily in dolomitic siltstone, but begins in an ~2 m thick dolomitic oolite member known as the Johnnie Oolite. The Johnnie Oolite is a consistent marker bed across the Death Valley region and has been characterized and discussed in many previous studies (Bergmann, Zentmyer, & Fischer, 2011; Corsetti & Kaufman, 2003; Kaufman, Corsetti, & Varni, 2007; Verdel et al., 2011). Fifteen samples from the Death Valley section were analyzed for U isotopes.

The precise stratigraphic/temporal correlation between different Shuram sections is difficult because of the lack of radiometric dates to directly constrain the initiation and termination of the Shuram excursion. Previous studies have variously suggested that the Shuram excursion is either a brief event occurring at ca. 560–550 Ma or a protracted event at ca. 580–550 Ma (see summary in Xiao et al., 2016). Thus, it is uncertain whether the initiation of the Shuram excursion temporally coincides or postdates the ca. 580 Ma Gaskiers glaciations (Pu et al., 2016), and it is also unclear whether the first appearance of diverse Ediacara‐type fossils in the Avalon assemblage at ca. 571 Ma (Pu et al., 2016) coincides or postdates the initiation of the Shuram excursion. Recent studies, however, suggest that the Shuram excursion was initiated around 580 Ma (Witkosky & Wernicke, 2018) and ended significantly earlier than 551 Ma (An et al., 2015; Xiao, Bykova, Kovalick, & Gill, 2017; Zhou et al., 2017). On the basis of a subsidence analysis of the Johnnie Formation that hosts the Shuram excursion, Witkosky and Wernicke (2018) concluded that the Shuram excursion occurred 585–579 Ma, thus overlapping with the ca. 580 Ma Gaskiers glaciations and predating all known Ediacara‐type fossils. In the Yangtze Gorges area of South China, Doushantuo Member III that hosts the Shuram excursion is separated from the ca. 551 Ma ash bed by strata that host two additional carbon isotope excursions (An et al., 2015; Zhou et al., 2017). Thus, the Shuram excursion may have ended significantly earlier than 551 Ma and may have lasted much less than 30 Myr as some previous studies suggested (Le Guerroué, Allen, Cozzi, Etienne, & Fanning, 2006). Regardless, recent paleomagnetic, rock magnetic, and cyclostratigraphic studies suggest that the Shuram excursion from different locations occurred synchronously (Gong, Kodama, & Li, 2017; Minguez & Kodama, 2017; Minguez, Kodama, & Hillhouse, 2015). For example, rock magnetic studies from globally separated sites—the Doushantuo Member III (EN3) in South China, the Wonoka Formation from the Flinders Ranges in South Australia, and the Johnnie Formation from the Death Valley, California, USA—suggest that the Shuram excursion at these localities is broadly synchronous over a duration of 8–10 Myr (Gong et al., 2017; Minguez & Kodama, 2017; Minguez et al., 2015). Ediacaran succession at the Bol'shoy Patom section in Siberia, one of the three sections in this study, is not constrained by radiometric and paleomagnetic data, and the largest negative δ^13^C_carb_ excursion is regarded as equivalent to the Shuram excursion found at other localities (e.g., Grotzinger et al., 2011; Melezhik, Fallick, & Pokrovsky, 2005; Melezhik et al., 2009).

## 3 ANALYTICAL METHODS

We have carefully selected fresh rock specimens to avoid veins and cleaned the specimens using 18.2 MΩ Milli‐Q water. The cleaned specimens were then dried and powdered to ~200 mesh using an agate ball mill. Approximately 5 g of each sample was dissolved in 1 M hydrochloric acid (HCl) for 24 hr at room temperature. Digests were centrifuged, and the supernatant was separated. Major, minor, and trace element concentrations were measured on a Thermo iCAP™ quadrupole inductively coupled plasma mass spectrometer (Q‐ICP‐MS) at the W. M. Keck Laboratory for Environmental Biogeochemistry at Arizona State University (ASU) on splits from each supernatant. Typical precision was better than 3% and 5% for major and trace elements, respectively, based on repeated analysis of in‐run check standards.

**Figure 2:**
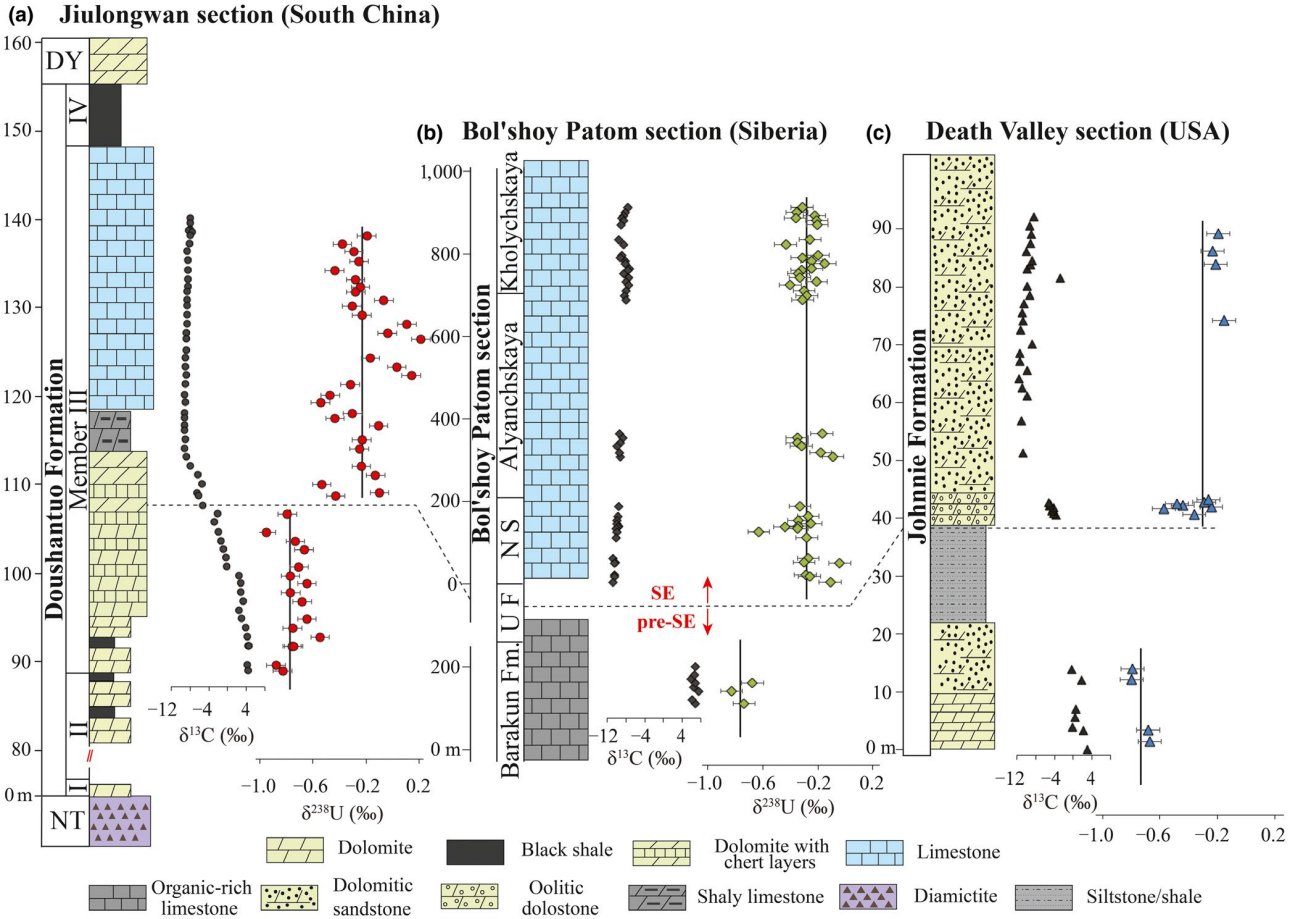
δ^238^U and δ^13^C_carb_ profiles of the three study sections. (a) Jiulongwan section in the Yangtze Platform, South China. (b) Bol'shoy Patom section, Siberia. (c) Death Valley section (the Johnnie Formation), western USA. Error bars of δ^238^U denote 2 standard derivations (2SD) and represent an average calculated from a large number of analyses of the CRM 145 standard (*n* = 200). DY, Dengying Formation; NS, Nikol'skaya Formation; NT, Nantuo Formation; UF, Urinskaya Formation. δ^13^C_carb_ profile of the Jiulongwan section is from Li et al. (2017). δ^13^C_carb_ profile of the Bol'shoy Patom section is from Melezhik et al. (2009). δ^13^C_carb_ profile of the Death Valley section is from Hardisty et al. (2017) [Colour figure can be viewed at http://wileyonlinelibrary.com]

Prior to U isotopes column chemistry, appropriate amounts of the ^236^U:^233^U double spike were added to each sample (e.g., Brennecka et al., 2011; Weyer et al., 2008; Zhang, Xiao, et al., 2018). The spike‐sample mixtures were evaporated to dryness and taken up in 3N HNO_3_. Uranium was purified using the UTEVA method for isotopic analysis (Brennecka et al., 2011; Chen et al., 2018; Kendall et al., 2015; Romaniello et al., 2013; Weyer et al., 2008; Zhang, Algeo, Cui, et al., 2019; Zhang, Algeo, Romaniello, et al., 2018; Zhang, Romaniello, et al., 2018; Zhang, Xiao, et al., 2018). All samples were put through UTEVA resin twice in order to completely remove matrix ions. The final purified U was dissolved in 0.32 M HNO_3_ and diluted to a U concentration of 50 ppb. Uranium isotopes were measured at ASU on a Thermo‐Finnigan Neptune multi‐collector ICP‐MS at low mass resolution. The standard solution CRM145 (50 ppb U) was analyzed every two samples. Two secondary standards CRM129a and Ricca ICP solution were measured after every fifteen measurements. Sample δ^238^U values were normalized by the average of the bracketing standards. The isotopic compositions of standards CRM145, CRM129a, and Ricca are 0.00 ± 0.07‰ (2 *SD*,* n* = 200), −1.74 ± 0.06‰ (2 *SD*,* n* = 30), and −0.28 ± 0.08‰ (2 *SD*,* n* = 30), respectively. The δ^238^U results are summarized in Figure 2 in the main text and in Data S1–S3.

## 4 URANIUM ISOTOPE RESULTS

An extremely negative δ^13^C_carb_ excursion that characterizes the SE is observed at each of the three sections studied here (Figure 2). At each section, δ^238^U shifts toward higher values as δ^13^C_carb_ declines during the onset of the SE (Figure 2). Samples immediately preceding the SE (pre‐SE) at Jiulongwan, at Bol'shoy Patom, and at Death Valley have remarkably consistent δ^238^U values of −0.74 ± 0.20‰ (2 *SD*, and hereafter, *n* = 16), −0.75 ± 0.15‰ (*n* = 3), and −0.73 ± 0.14‰ (*n* = 4), respectively (Figure 2). Samples deposited during the SE at Jiulongwan, Bol'shoy Patom, and Death Valley again are consistent, with δ^238^U values of −0.23 ± 0.38‰ (*n* = 30), −0.28 ± 0.20‰ (*n* = 42), and −0.31 ± 0.31‰ (*n* = 11), respectively (Figure 2). Averaged over all three sections, δ^238^U values of the SE carbonates (−0.26 ± 0.29‰) are significantly higher than those of pre‐SE carbonates (−0.74 ± 0.17‰, *p* < .0001) but are only slightly lower than modern Bahamian carbonates (−0.12 ± 0.28‰, 2 *SD*) (Chen et al., 2018).

## 5 EVIDENCE FOR PRIMARY OCEANOGRAPHIC SIGNALS

### 5.1 Post‐depositional diagenetic alteration

We compared our U isotope data to standard carbonate diagenetic indicators, such as Mn/Sr ratios and O isotope compositions, to evaluate the influence of post‐depositional burial diagenesis. We note that these traditional diagenetic proxies are not explicitly developed for U isotopes but carbonate C, O, and Sr isotope systematics (e.g., Chen et al., 2018). Although these proxies have their limitations and may not be directly relevant to evaluate diagenesis for carbonate U isotopes proxy, numerical modeling of diagenetic rock‐fluid interactions suggests that δ^238^U should be more robust against diagenetic fluid exchange than δ^18^O and ^87^Sr/^86^Sr (Chen et al., 2018; Lau, Macdonald, Maher, & Payne, 2017). Thus, these traditional carbonate diagenetic indicators may be useful in identifying samples with diagenetically altered δ^238^U signatures.

**Figure 3:**
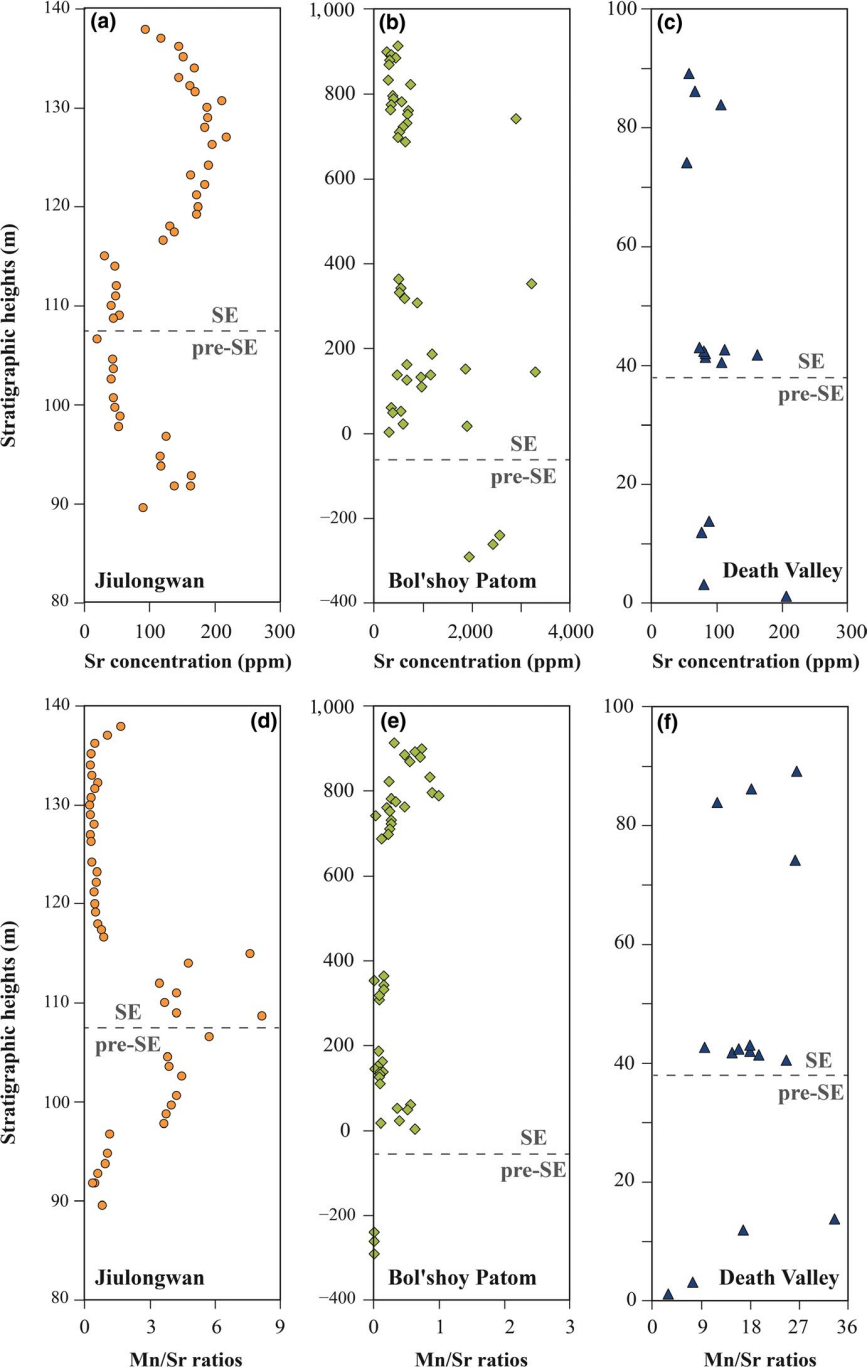
Stratigraphic variations in Sr concentrations and Mn/Sr ratios from the Jiulongwan section (a and d), the Bol'shoy Patom section (b and e), and the Death Valley section (c and f) [Colour figure can be viewed at http://wileyonlinelibrary.com]

Mn/Sr ratios in carbonate precipitates have commonly been used as indicators of post‐depositional alteration (e.g., Gilleaudeau, Sahoo, Kah, Henderson, & Kaufman, 2018; Jacobsen & Kaufman, 1999; Veizer, 1989), with a cutoff of 3–10 suggested for Precambrian carbonate sedimentary rocks (e.g., Gilleaudeau et al., 2018; Jacobsen & Kaufman, 1999). The Mn/Sr ratios of Jiulongwan carbonates range between 0.27 and 8.16, with 32 out of 49 showing Mn/Sr ratios <3, indicating that these carbonates are generally well preserved (Figure 3). The Mn/Sr ratios of Bol'shoy Patom carbonates range between 0 and 0.99, indicating that they are exceptionally well preserved (Figure 3). The Mn/Sr ratios of Johnnie sediments range between 2.79 and 35.46 (Figure 3). The relatively higher Mn/Sr ratios may attribute to lithological differences in the Johnnie sediment relative to the other two study sections. The Johnnie Formation is mainly comprised of dolomitic sandstones, which have a low capacity to reserve Sr but a high capacity to reserve Mn, thus having relatively high Mn/Sr ratios (Gilleaudeau et al., 2018; Veizer, 1983). We further investigated the geochemical correlations of Mn/Sr–δ^238^U, Mn/Sr–U concentration, Sr concentration–δ^238^U, Sr concentration–U concentration, Mn concentration–δ^238^U, and Mn concentration–U concentration for all study samples; neither δ^238^U nor U concentration shows statistically systematic correlations with Mn/Sr, Sr concentration, or Mn concentration (Tables 1 and 2).

**Table 1:** Linear regression coefficients (*multiple R)* and the associated *p*‐values calculated to test the influence of diagenetic indicators on δ^238^U

	*multiple R*	*p*‐value
South China section		
δ^238^U versus δ^13^C	.64	<.01
δ^238^U versus Mg/Ca (mol:mol)	.26	<.01
δ^238^U versus Sr/Ca (ppm/w.t.%)	.08	.05
δ^238^U versus δ^18^O	.22	<.01
U concentration versus δ^18^O	.01	.64
δ^238^U versus Sr concentration	.24	<.01
δ^238^U versus Mn concentration	.04	.18
δ^238^U versus Mn/Sr	.08	.07
δ^238^U versus Rb/Sr	.00	.74
δ^238^U versus U concentration	.14	.01
δ^238^U versus Th/U	.09	.04
δ^238^U versus U/Al (ppm/w.t.%)	.01	.62
δ^238^U versus Mo/U ratios	.07	.95
Siberia section		
δ^238^U versus δ^13^C	.57	<.01
δ^238^U versus Mg/Ca (mol:mol)	.05	.12
δ^238^U versus Sr/Ca (ppm/w.t.%)	.15	<.01
δ^238^U versus δ^18^O	.11	.03
U concentration versus δ^18^O	.15	.01
δ^238^U versus Sr concentration	.19	<.01
δ^238^U versus Mn concentration	.20	<.01
δ^238^U versus Mn/Sr	.16	<.01
δ^238^U versus Rb/Sr	.07	.08
δ^238^U versus U concentration	.04	.17
δ^238^U versus Th/U	.01	.42
δ^238^U versus U/Al (ppm/w.t.%)	.03	.24
δ^238^U versus Mo/U ratios	.00	.44
Death Valley section		
δ^238^U versus δ^13^C	.80	<.01
δ^238^U versus Mg/Ca (mol:mol)	.01	.75
δ^238^U versus Sr/Ca (ppm/w.t.%)	.01	.73
δ^238^U versus δ^18^O	.41	.02
U concentration versus δ^18^O	.03	.58
δ^238^U versus Sr concentration	.03	.54
δ^238^U versus Mn concentration	.00	.96
δ^238^U versus Mn/Sr	.01	.69
δ^238^U versus Rb/Sr	.37	.02
δ^238^U versus U concentration	.06	.39
δ^238^U versus Th/U	.24	.06
δ^238^U versus U/Al (ppm/w.t.%)	.39	.01
δ^238^U versus Mo/U ratios	.02	.63

Note

*p*‐value <.05 denotes the regression statistical analyses between two variables are statistically significant; *p*‐value >.05 denotes the regression statistical analyses between two variables are not statistically significant.

**Table 2:** Linear regression coefficients (*multiple R)* and the associated *p*‐values calculated to test the influence of diagenetic indicators on U concentrations

	*multiple R*	*p*‐value
South China section		
U concentration versus δ^13^C	.38	<.01
U concentration versus Mg/Ca (mol:mol)	.29	.04
U concentration versus Sr/Ca (ppm/w.t.%)	.21	.14
U concentration versus δ^18^O	.08	.57
U concentration versus Sr concentration	.25	.09
U concentration versus Mn concentration	.25	.08
U concentration versus Mn/Sr	.17	.23
U concentration versus Rb/Sr	.05	.74
U concentration versus Mo concentration	.60	<.01
Siberia section		
U concentration versus δ^13^C	.24	.12
U concentration versus Mg/Ca (mol:mol)	.23	.15
U concentration versus Sr/Ca (ppm/w.t.%)	.58	<.01
U concentration versus δ^18^O	.30	.06
U concentration versus Sr concentration	.63	<.01
U concentration versus Mn concentration	.33	.03
U concentration versus Mn/Sr	.41	<.01
U concentration versus Rb/Sr	.46	<.01
U concentration versus Mo concentration	.05	<.01
Death Valley section		
U concentration versus δ^13^C	.27	.26
U concentration versus Mg/Ca (mol:mol)	.16	.48
U concentration versus Sr/Ca (ppm/w.t.%)	.13	.58
U concentration versus δ^18^O	.11	.44
U concentration versus Sr concentration	.17	.47
U concentration versus Mn concentration	.14	.59
U concentration versus Mn/Sr	.06	.81
U concentration versus Rb/Sr	.26	.26
U concentration versus Th/U	.26	.26
U concentration versus Mo concentration	.34	.14

Note

*p*‐value <.05 denotes the regression statistical analyses between two variables are statistically significant; *p*‐value >.05 denotes the regression statistical analyses between two variables are not statistically significant.

Several researchers have argued, on the basis of positive correlations between carbon and oxygen isotope ratios, that the SE carbonates may have undergone extensive post‐depositional alteration (e.g., Derry, 2010; Grotzinger et al., 2011; Knauth & Kennedy, 2009). Chen et al. (2018) observed that meteoritic diagenesis of Bahamian carbonate likely led to a ~0.2‰ enrichment of δ^238^U in altered carbonates compared to samples that only experienced marine phreatic or marine burial diagenesis. To test whether a similar process could have impacted SE carbonates, we investigated the extent of correlation of δ^238^U and U concentrations with δ^18^O for our samples (Tables 1 and 2). We did not observe any systematic correlations between δ^18^O and [U] for any of the three sections (Jiulongwan, *R* ^2^ = .01; Bol'shoy Patom, *R* ^2^ = .14; Death Valley, *R* ^2^ = .03). Likewise, we did not observe systematic correlations between δ^18^O and δ^238^U for the Jiulongwan and the Bol'shoy Patom sections (*R* ^2^ = .19 and *R* ^2^ = .11, respectively). However, we did observe a weak‐to‐moderate correlation between δ^18^O and δ^238^U for the Death Valley section (*R* ^2^ = .41), possibly indicating meteoric alteration of δ^238^U at this section. Although it is difficult to entirely rule out a meteoritic diagenetic influence, we argue that the relatively weak correlations between δ^18^O and δ^238^U, large magnitude of the δ^238^U shift (0.5‰), and strong consistency of δ^238^U between widely spaced sections argue against a meteoritic diagenetic origin for the observed uranium isotope trends and instead strongly favor a primary seawater origin.

In carbonates that underwent extensive recrystallization, δ^238^U may be offset from primary depositional values, and therefore, petrographic studies and duplication in different sections are necessary when studying carbonate δ^238^U (e.g., Hood, 2016). Prior studies from the same outcrops sampled in this study suggest that the Bol'shoy Patom samples and the Jiulongwan limestones typically preserve pristine sedimentary fabrics such as microbially laminated micrites, while the Jiulongwan and the Johnnie dolostones preserve relative fine‐grained, planar structures [see petrographic photographs for the study samples in McFadden et al. (2008) and Melezhik et al. (2009)]. These petrographic observations, together with the consistent δ^238^U signatures at three paleogeographically widely separated sections that have deposited under different water depths and have experienced completely different diagenetic histories, strongly suggest that δ^238^U was not systematically altered by diagenesis.

We note that some carbonates at the Jiulongwan section have comparatively high δ^238^U values, which likely reflects incorporation of ^238^U‐enriched U(IV) from local anoxic porewaters during early diagenesis (Romaniello et al., 2013; discussed further below). Prior Fe‐S‐C systematic and Ce anomaly studies have suggested that the local depositional environment at the Jiulongwan section was anoxic (Li et al., 2010; Ling et al., 2013). Nevertheless, although minor diagenetic influence on the δ^238^U of individual samples is unavoidable, the lack of statistical correlation between our δ^238^U data and geochemical indicators of diagenesis suggests that the δ^238^U recorded by SE carbonates was not pervasively nor systematically altered. Importantly, our key interpretation is built on the average of the three study sections rather than the Jiulongwan section alone.

### 5.2 Evaluation of detrital contamination

**Figure 4:**
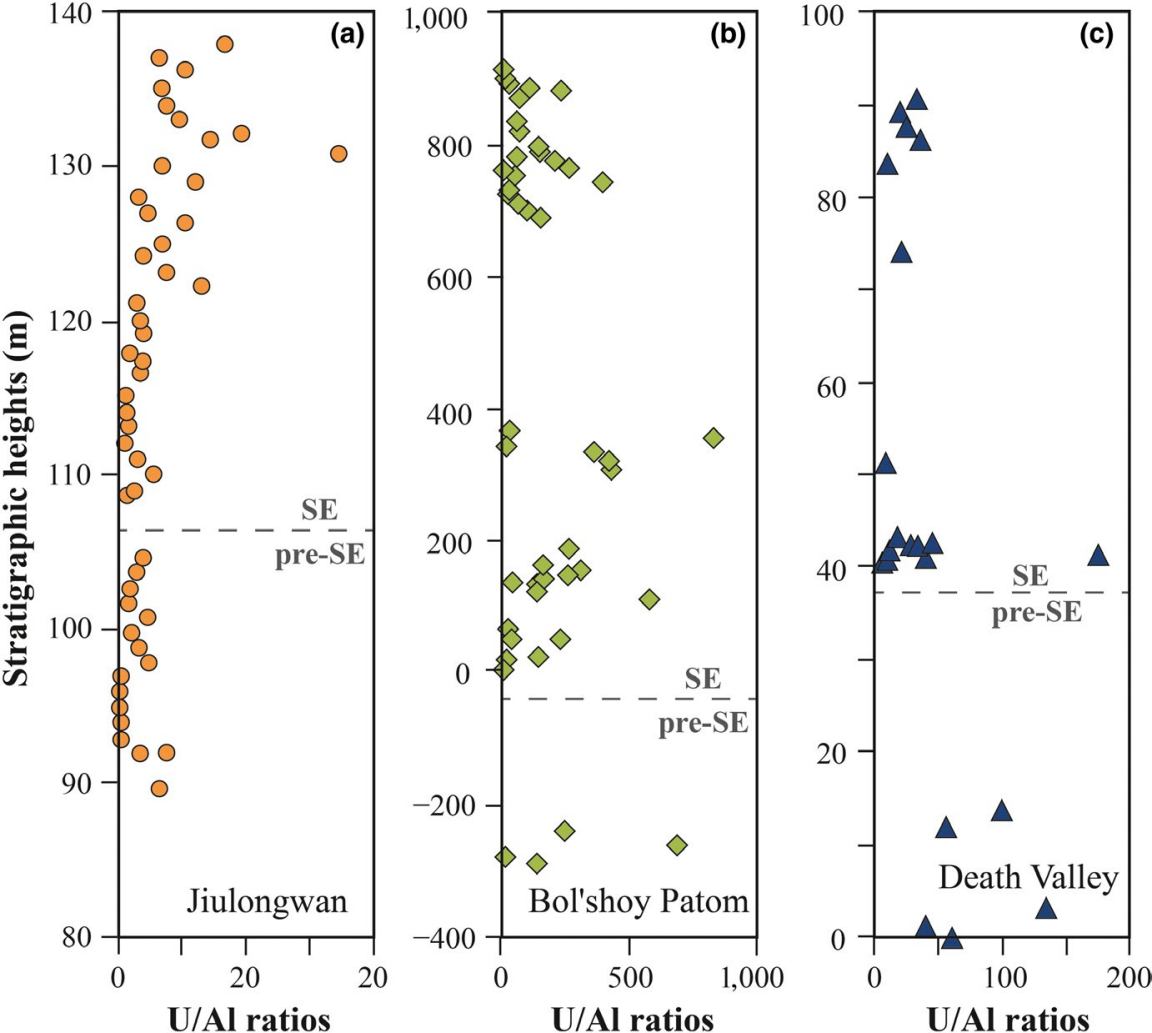
Stratigraphic variations in U/Al ratios from the Jiulongwan section (a), the Bol'shoy Patom section (b), and the Death Valley section (c) [Colour figure can be viewed at http://wileyonlinelibrary.com]

Changes in the extent of detrital input might also cause a δ^238^U offset. Our samples were dissolved in 1 M hydrochloric acid (HCl) prior to extraction of U, which will minimize dissolution of any non‐carbonate minerals (e.g., silicates) and organic matter. This expectation is supported by the overall high U/Al ratios in our analyses. The average U/Al ratios for the in the upper continental crust and the topsoil are ~0.33 ppm/wt.% (Rudnick & Gao, 2014) and 0.58 ± 1.13 ppm/wt.% (Cole, Zhang, & Planavsky, 2017), respectively; U/Al ratios in our samples are substantially enriched above crustal value or topsoil mean value by about 1 ~ 3 orders of magnitude (Figure 4), indicating that our dissolution protocol had efficiently extracted carbonate bounded U, and thus, the majority of U in the samples is authigenic rather than detrital in origin. Although there is a moderate correlation between δ^238^U and U/Al ratio at the Death Valley section, neither U/Al ratio nor U concentration shows statistically systematic correlations with δ^238^U at the Jiulongwan and the Siberia sections (Tables 1 and 2), indicating that observed δ^238^U trends are not related to detrital influence.

### 5.3 Evaluation of influence of dolomitization on δ^238^U

**Figure 5:**
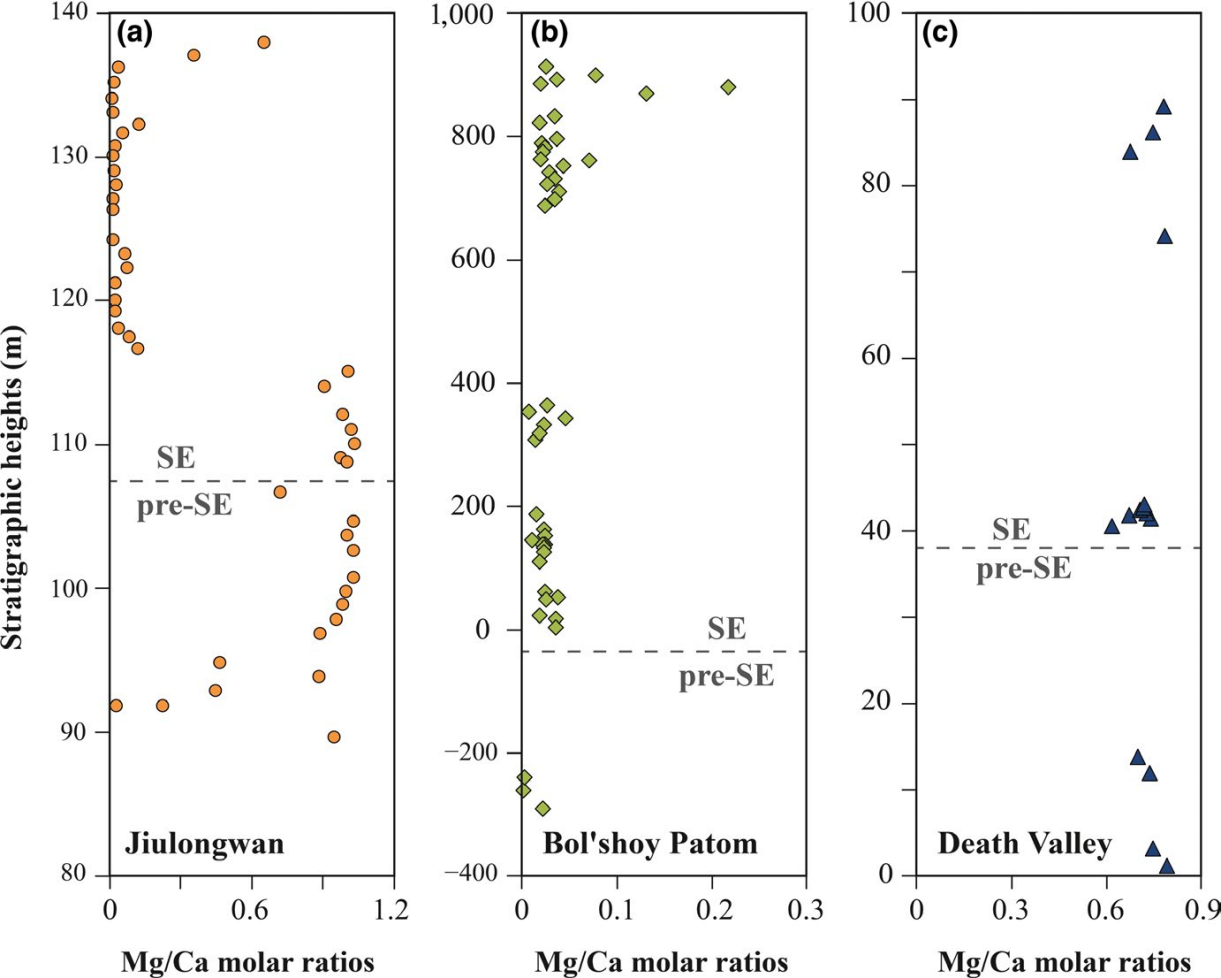
Stratigraphic variations in Mg/(Mg+Ca) ratios and Mg/(Mg+Ca) ratios versus δ^238^U plots. (a) and (d) are from the Jiulongwan section, (b) and (e) are from the Bol'shoy Patom section, and (c) and (f) are from Death Valley section [Colour figure can be viewed at http://wileyonlinelibrary.com]

Two independent lines of evidence document that lithological changes (e.g., dolomitization) are unlikely a significant contributor to the observed shift in δ^238^U across the Shuram event. First, although the shift toward heavier δ^238^U values approximately coincides with a lithological change from dolostone to limestone at the Jiulongwan section, the onset of the positive δ^238^U excursion in the Johnnie Formation occurs in an oolitic dolomite unit, it does not coincide with any lithological changes, and positive δ^238^U values continue in an overlying dolomitic sandstone unit (Hardisty et al., 2017). Stratigraphic variations in Mg/Ca molar ratios document no lithological changes at the Bol'shoy Patom section (Figure 5), which is mainly comprised of well‐preserved limestone with high‐Sr concentrations (Melezhik et al., 2009).

Second, δ^238^U studies from both modern Bahamian carbonate sediments and Permian–Triassic carbonates strongly suggest that dolomitization does not appear to be a major issue for paleo‐δ^238^U records. Romaniello et al. (2013) observed δ^238^U changes associated with dolomitization in a modern Bahamian tidal pond, as reflected in a strong correlation of δ^238^U with Mg/Ca (*R* ^2^ = .96, *p* < .001). However, subsequent work by Chen et al. (2018) and Tissot et al. (2018), who revisited the dolomitization question with a larger sample set from several cores through the Bahamian carbonate platform, shows no statistically significant differences between calcite and dolomite. There are now δ^238^U data from seven widely spaced carbonate sections spanning the Permian–Triassic boundary [the Dajiang (Lau et al., 2016), Guandao (Lau et al., 2016), Dawen (Brennecka et al., 2011), and Daxiakou (Elrick et al., 2017) sections in South China; the Taşkent section in Turkey (Lau et al., 2016); the Zal section in Iran (Zhang, Romaniello, et al., 2018); and the Kamura section in Japan (Zhang, Algeo, Romaniello, et al., 2018)]. All of these sections show strikingly similar trends in δ^238^U across the Permian–Triassic boundary, which is remarkable because they span 1,000s of km—even in different ocean basins—and have experienced very different diagenetic histories, including dolomitization. Additionally, we also investigated the extent of correlation between Mg/Ca molar ratios and δ^238^U for our SE samples. No statistically significant correlations are observed (Table 1), suggesting that the influence of lithology on δ^238^U is not significant. Furthermore, as with the Permian–Triassic δ^238^U work (Zhang, Algeo, Romaniello, et al., 2018), we focus in this manuscript on the comparison of multiple widely spaced Shuram sections, which come from different continents and different water depths, and experienced different diagenetic histories. Similar to the Permian–Triassic studies (Zhang, Algeo, Romaniello, et al., 2018), the three widely separated sections with very different lithologies yielded largely identical δ^238^U records, which strongly argue against anything but primary oceanographic trends.

### 5.4 Isotopic offset induced from syndepositional diagenesis

Modern carbonate sediments have a δ^238^U composition that is 0.2–0.4‰ higher than that of the contemporaneous seawater (0.27‰ by average; Chen et al., 2018; Romaniello et al., 2013; Tissot et al., 2018), which likely reflects incorporation of ^238^U‐enriched U(IV) from anoxic porewaters during early diagenesis or variation in porewater U‐speciation during carbonate recrystallization. Syndepositional diagenesis of carbonates occurs because shallow, relatively permeable carbonates can sequester dissolved U(VI) from the overlying oxic water via advective and diffusive transport. This semi‐open system behavior allows the exchange of U isotopes and can lead to slight ^238^U enrichment in bulk carbonates (Chen et al., 2018; Romaniello et al., 2013; Tissot et al., 2018). However, this process does not operate at greater burial depths as the mobility of U is severely restricted in anoxic porewaters, as shown by near‐zero porewater U concentrations in deep Bahamian drillcores (Henderson, Slowey, & Haddad, 1999). On this basis, we applied a diagenetic correction factor of 0.2‰–0.4‰ to the measured δ^238^U values prior to U isotope mass balance calculations presented below. Considering this range of diagenetic offset, our best estimates of δ^238^U for the pre‐SE and SE seawaters are −0.94‰ to −1.14‰ and −0.46‰ to −0.66‰, respectively.

## 6 STRATIGRAPHIC VARIATION OF U CONCENTRATIONS

Several previous U isotope studies suggested that in unaltered rocks, changes to the extent of global seafloor oxygenation will affect the dissolved seawater reservoir of U, and in return the abundance of U incorporated into marine carbonates (Brennecka et al., 2011; Elrick et al., 2017; Lau et al., 2016). Under ideal conditions, stratigraphic variation in U concentrations can record meaningful seawater redox variations, but this relationship can be easily masked by other sources of variation (e.g., Lau et al., 2017). Notably, prior studies have shown that the distribution coefficient of U into aragonite is significantly larger than for calcite (DeCarlo, Gaetani, Holcomb, & Cohen, 2015; Meece & Benninger, 1993; Reeder, Nugent, Lamble, Tait, & Morris, 2000). For instance, an experimental study showed that the partition coefficients for U in aragonite range from 1.8 to 9.8, while the partition coefficient for U in calcite is <0.2 and may be as low as 0.046 (Meece & Benninger, 1993). Thus, environmental and ecological changes that drive variations in the abundance of primary aragonite and calcite have a large effect on sediment U concentration. For example, the modern Bahamian carbonates have U concentrations that vary from <0.1 to >4 ppm but their δ^238^U values are the same (Romaniello et al., 2013).

In contrast, the effect of mineralogy and carbonate ion concentration on δ^238^U is more limited. Uranium isotope measurements of aragonite and high‐Mg calcite primary precipitates exhibit no offset from seawater (Romaniello et al., 2013). Laboratory‐precipitated calcite and aragonite at pH ∼8.5 showed only minor (<0.13‰) fractionation between the liquid medium and the solid (Chen, Romaniello, Herrmann, Wasylenki, & Anbar, 2016; Stirling, Andersen, Warthmann, & Halliday, 2015). At pH ∼7.5, the precipitates of both polymorphs exhibit no fractionation (Chen et al., 2016). Therefore, changing carbonate mineralogy can result in large differences in uranium concentrations but only small changes in the isotopic composition (Lau et al., 2017).

**Figure 6:**
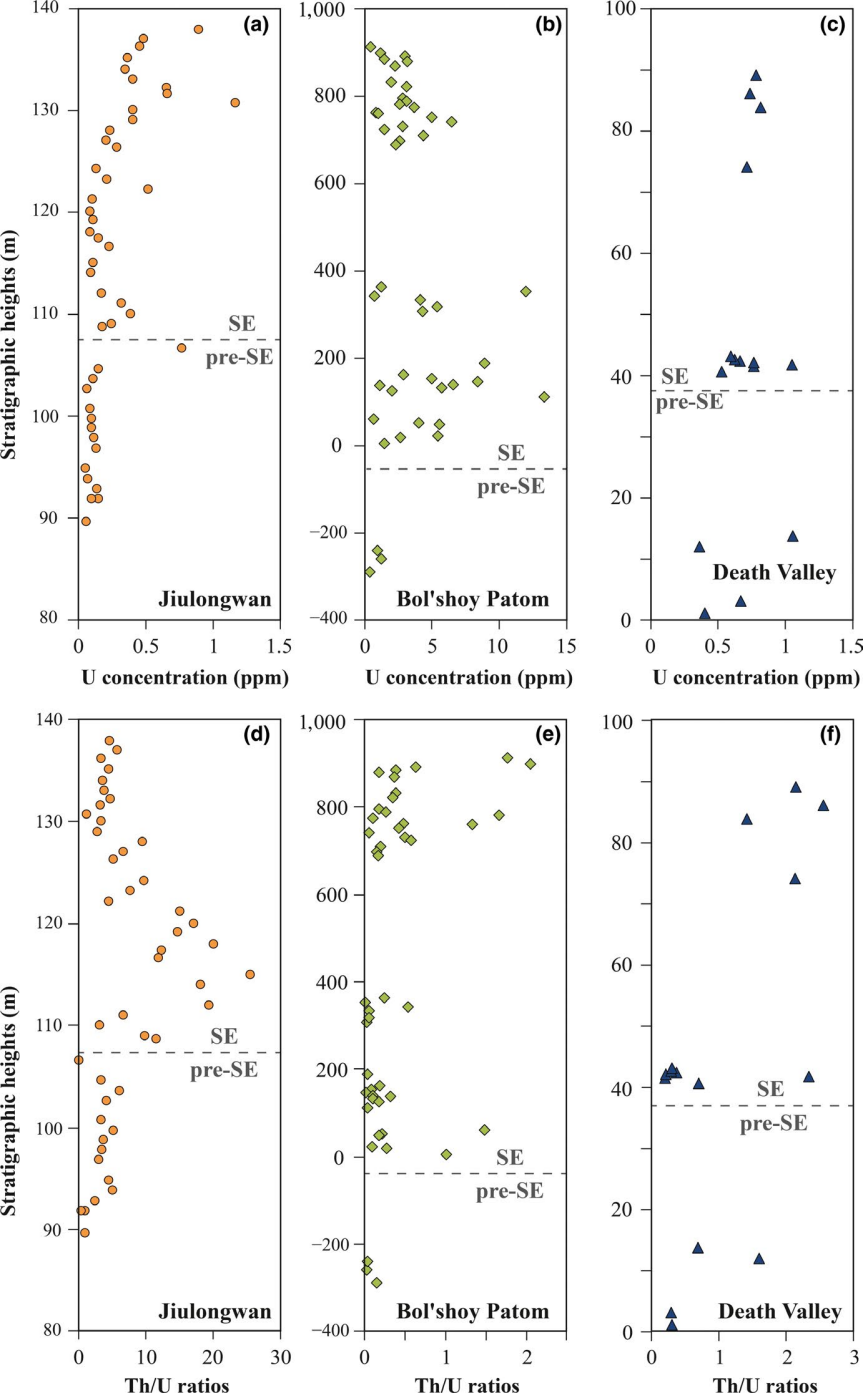
Stratigraphic variations in U concentration and Th/U ratios. (a) and (d) are from the Jiulongwan section, (b) and (e) are from the Bol'shoy Patom section, and (c) and (f) are from the Death Valley section [Colour figure can be viewed at http://wileyonlinelibrary.com]

There are obvious lithological and mineralogical changes in the studied sections. For example, the Jiulongwan section is composed of interlayered limestone and dolostone, and the Death Valley section is composed of dolomitic sandstone, sandy dolostone, and dolostone, while the Siberian section is composed of well‐preserved high‐Sr limestone. Some of these limestones may originally be aragonite and/or high‐Mg calcite (e.g., the high‐Sr carbonates from Siberia; Melezhik et al., 2009). The Jiulongwan section and the Siberian sections have very different U concentrations whereas their U isotope trends are the same. Most of samples (44 out of 49 samples) from Jiulongwan have U concentrations <0.5 ppm. In contrast, 25 of the 44 samples from the Siberia have U concentration >3 ppm, with some samples having U > 10 ppm (Figure 6). There are also relatively strong correlations between U concentrations and Sr‐based indicators (Sr concentration, Sr/Ca ratio, Mn/Sr ratio, and Rb/Sr ratio) at the Siberia section (Table 2), further confirming a mineralogical control on the U concentration. We thus hypothesize that the decoupling of U concentration from δ^238^U in the study sections can be attributed to mineralogical/lithological shifts that affect only the reliability of the carbonate U concentration paleoredox proxy and but not δ^238^U. We therefore focus on the δ^238^U data as a paleoredox proxy, although we acknowledge that the mechanisms that led to the differences in U concentration merit further investigation.

## 7 NEAR‐MODERN LEVELS OF OCEAN OXYGENATION DURING THE SHURAM EVENT

**Figure 7:**
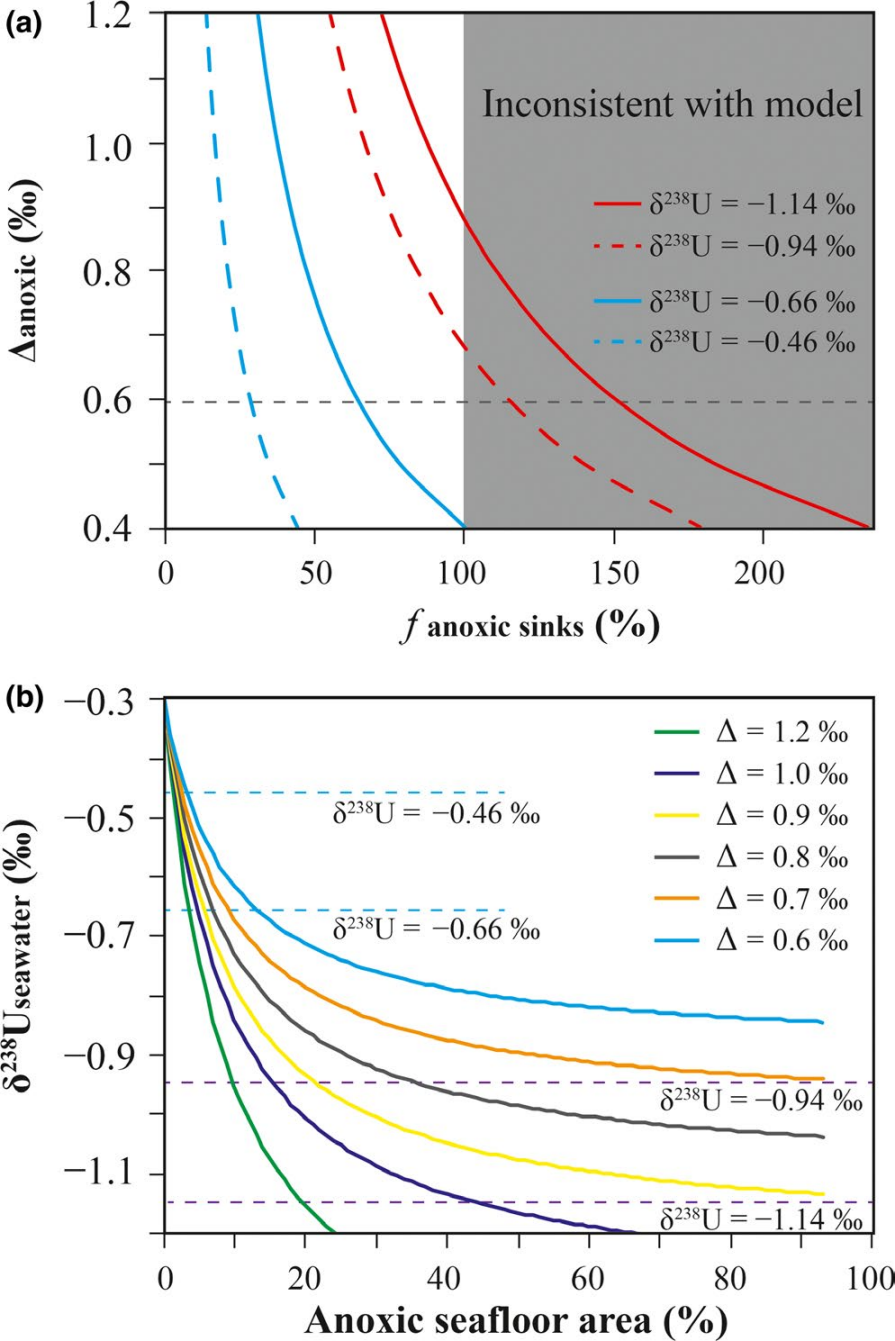
Steady‐state U isotope mass balance model results. (a) The fraction of oceanic U inputs removed into anoxic/euxinic sediments (horizontal axis) varies as a function of the fractionation factor (Δ_anoxic_; vertical axis) between seawater and anoxic/euxinic sediments. (b) Steady‐state U isotope mass balance calculations show variations of seawater δ^238^U values as a function of anoxic seafloor area (%), keeping suboxic seafloor area fixed at 0% of total seafloor area and testing the sensitivity to possible Δ_anoxic_ values. In reality, suboxic seafloor area would co‐vary with anoxic/euxinic seafloor area; thus, this modeling exercise gives us the lowest estimation of anoxic/euxinic seafloor area [Colour figure can be viewed at http://wileyonlinelibrary.com]

Because the duration of the SE (>8 Myr; Minguez & Kodama, 2017) is significantly longer than the residence time of U in the SE ocean (<0.5 Myr), we use a simple steady‐state isotopic mass balance model combined with the measured δ^238^U data to assess changes in the size of the anoxic U sink and the implications for the areal extent of anoxic bottom waters (see Zhang, Xiao, et al., 2018 for details):

(1) $$\delta^{238}U_{\text{input}}=\left(f_{\text{anoxic}}\times \delta^{238}U_{\text{anoxic}}\right)+\left(f_{\text{other}}\times \delta^{238}U_{\text{other}}\right)$$

(2) $$\begin{array}{c} \delta^{238}U_{\text{seawater}}\\ =\delta^{238}U_{\text{input}}-\frac{A_{\text{anoxic}}*k_{\text{anoxic}}*\Delta_{\text{anoxic}}+\left(A_{\text{ocean}}-A_{\text{anoxic}}\right)*k_{\text{other}}*\Delta_{\text{other}})}{A_{\text{anoxic}}*k_{\text{anoxic}}+\left(A_{\text{ocean}}-A_{\text{anoxic}}\right)*k_{\text{other}}} \end{array}$$

where δ^238^U_input_, δ^238^U_anoxic_, and δ^238^U_other_, and δ^238^U_seawater_ denote the U isotopic compositions of riverine input, anoxic sink, all other sedimentary sinks, and seawater, respectively. The variables *f*_anoxic_ and *f*_other_ represent the fraction of total U removed to the respective sinks, Δ_anoxic_ and Δ_other_ represent the isotope fractionation factor between seawater and the respective sinks, *A*_anoxic_ and *A*_ocean_ denote anoxic seafloor area and seafloor area of the world ocean, and *k*_anoxic_ and *k*_other_ represent the area‐weighted first‐order removal rate constants for each of the respective sinks. The modeling outputs are given in Figure 7.

This model indicates that in order to account for pre‐SE seawater δ^238^U as low as −0.94‰ to −1.14‰, large areas of seafloor must have been overlain by anoxic waters. The precise extent of ocean anoxia calculated from the mass balance model (Equation 2) is sensitive to Δ_anoxic_ values (Lau et al., 2017; Zhang, Xiao, et al., 2018). Assuming Δ_anoxic_ = 0.6‰—an average value that is representative of modern anoxic basins like the Saanich Inlet (Holmden, Amini, & Francois, 2015) and the Black Sea (Andersen et al., 2014), the δ^238^U data imply that nearly 100% of the total U ocean sink in the pre‐Shuram ocean was accounted for by removal into anoxic sediments. If we consider a range of plausible fractionation factors between 0.6‰ and 0.85‰ (Yang, Kendall, Lu, Zhang, & Zheng, 2017; Zhang, Xiao, et al., 2018), representing the range of estimates inferred from modern analogs and microbial U reduction experiments, the estimated area of anoxic seafloor in the pre‐SE ocean ranges from 26% to 100% (Figures 2 and 7). Thus, compared with the modern ocean which has ~0.11% anoxic seafloor (e.g., Sheen et al., 2018), widespread anoxia in the pre‐SE ocean is implicated for all plausible values of Δ_anoxic_.

During the SE, the marked positive shift in δ^238^U_seawater_ to values of −0.46‰ to −0.66‰ corresponds to a dramatic expansion of seafloor oxygenation. The extent of ocean anoxia inferred from these values is also sensitive to Δ_anoxic_ values. However, the majority of seafloor needed to be oxic to drive SE seawater δ^238^U to higher values between −0.46‰ and −0.66‰. Under all circumstances, the calculated anoxic seafloor area in the SE ocean is <6% (Figures 2 and 7). Thus, the SE represents a significant ocean oxygenation event, and such a rapid increase in global ocean oxygenation likely occurred within 0.4 Myr if we accept a duration of the SE of ~8 Myr and assume a constant sedimentation rate during the SE event (Minguez & Kodama, 2017).

The new δ^238^U data reinforce previous studies that argued for an oceanic oxygenation event during the SE (Canfield et al., 2007; Fike et al., 2006; Hardisty et al., 2017; McFadden et al., 2008). Organic‐rich mudrocks deposited near the end of the SE have high δ^238^U values that point to an episode of extensive oceanic oxygenation ca. 560–551 Myr ago (Kendall et al., 2015), consistent with the δ^238^U data presented here suggesting that the SE represents a significant ocean oxygenation event. Furthermore, seemingly conflicting results from Fe‐S‐C data suggesting local oxygenation and local sustained anoxia as well as local redox stratification (Canfield et al., 2008; Johnston et al., 2013; Li et al., 2010; Sahoo et al., 2016; Sperling et al., 2015) can be reconciled if the ocean redox regime during the SE was similar to or slightly more reducing than the present day. Specifically, this would imply generally oxic global ocean conditions coexisting with anoxia in some local shelf settings (such as oxygen minimum zones) and semi‐enclosed basins (such as the modern Cariaco Basin). The combined U proxy from this study and Fe‐S‐C proxies from previously published studies ultimately provide a more detailed illustration of the redox state of the ocean on global and local scales.

## 8 GLOBAL MARINE REDOX CHANGE DROVE THE RISE AND FALL OF THE EDIACARA BIOTA

**Figure 8:**
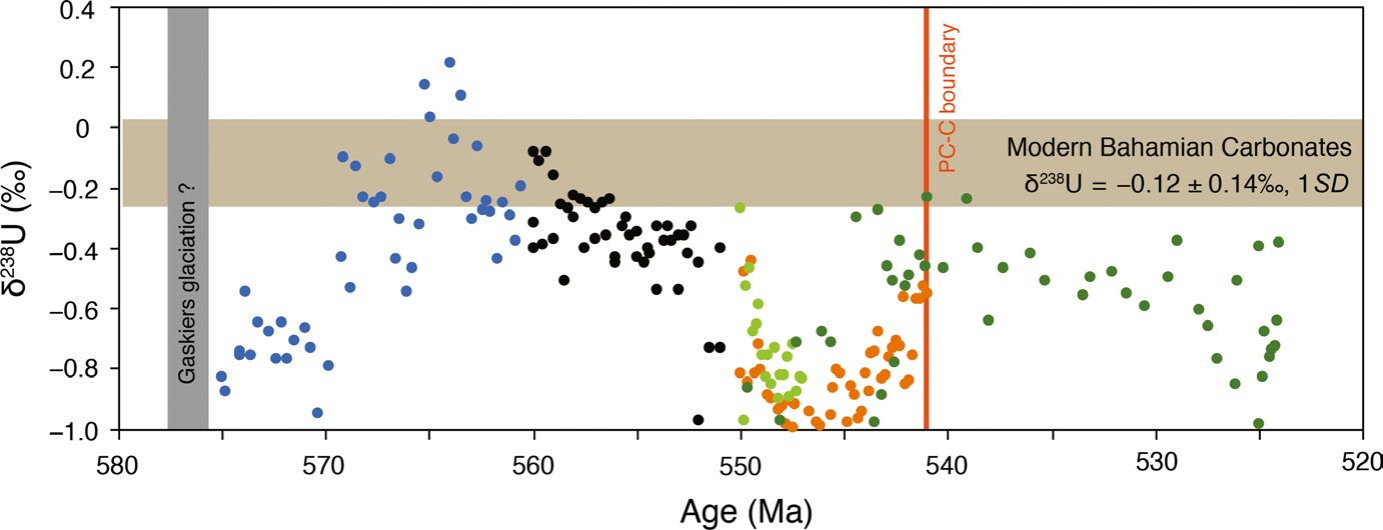
Compilation of Ediacaran–Cambrian δ^238^U data. Data sources: This study (light blue); Kendall et al., 2015 (black); Zhang, Xiao, et al., 2018 (orange); Wei et al., 2018 (dark green); Tostevin et al., 2019 (light green) [Colour figure can be viewed at http://wileyonlinelibrary.com]

The U isotope data from this study combined with previously published Ediacaran and Early Cambrian U isotope data yield a complex picture of oscillatory ocean redox conditions at the Ediacaran−Cambrian transition (Figure 8). The present study suggests that the SE represents a significant global deep ocean oxygenation event, consistent with a recent U isotope study that suggests an episode of extensive ocean oxygenation ca. 560 to 551 Myr ago (Kendall et al., 2015). U isotope studies from the terminal Ediacaran to the early Cambrian provide evidence for widespread anoxic conditions during the terminal Ediacaran Period (ca. 551 − 541 Ma) and in the early Cambrian (during the Cambrian Age 2 at ca. 525 Ma) with a temporary transition to more oxygenated conditions at the Ediacaran−Cambrian boundary (Tostevin et al., 2019; Wei et al., 2018; Zhang, Xiao, et al., 2018). Therefore, this and previous studies confirm that the oceanic redox evolution from the Neoproterozoic to the Paleozoic was not a history of simple and unidirectional oxygenation, but one with rapid perturbations in the relative proportions of anoxic versus oxic waters (Figure 8) (Johnston et al., 2013; Sahoo et al., 2016; Wood et al., 2015; Zhang, Xiao, et al., 2018).

The possible causal relationship between oxygenation events and early animal evolution is a topic of broad interest. Molecular clock and sponge biomarker studies suggest that multicellular animals diverged in the Cryogenian Period (~720–635 Myr ago) or earlier (Cunningham et al., 2017; Sperling and Stockey, 2018; Zumberge et al., 2018, but see Nettersheim et al., 2019). However, Cryogenian animals may have been morphologically simple sponge‐like creatures and likely required little oxygen (Mills, Lenton, & Watson, 2014; Sperling et al., 2013). Macroscopic and morphologically complex animals that engaged in energetically expensive lifestyles such as mobility likely required more oxygen, and these animals did not appear in the fossil record until the late Ediacaran Period, as represented by certain taxa in the Ediacara biota (Xiao & Laflamme, 2009).

**Figure 9:**
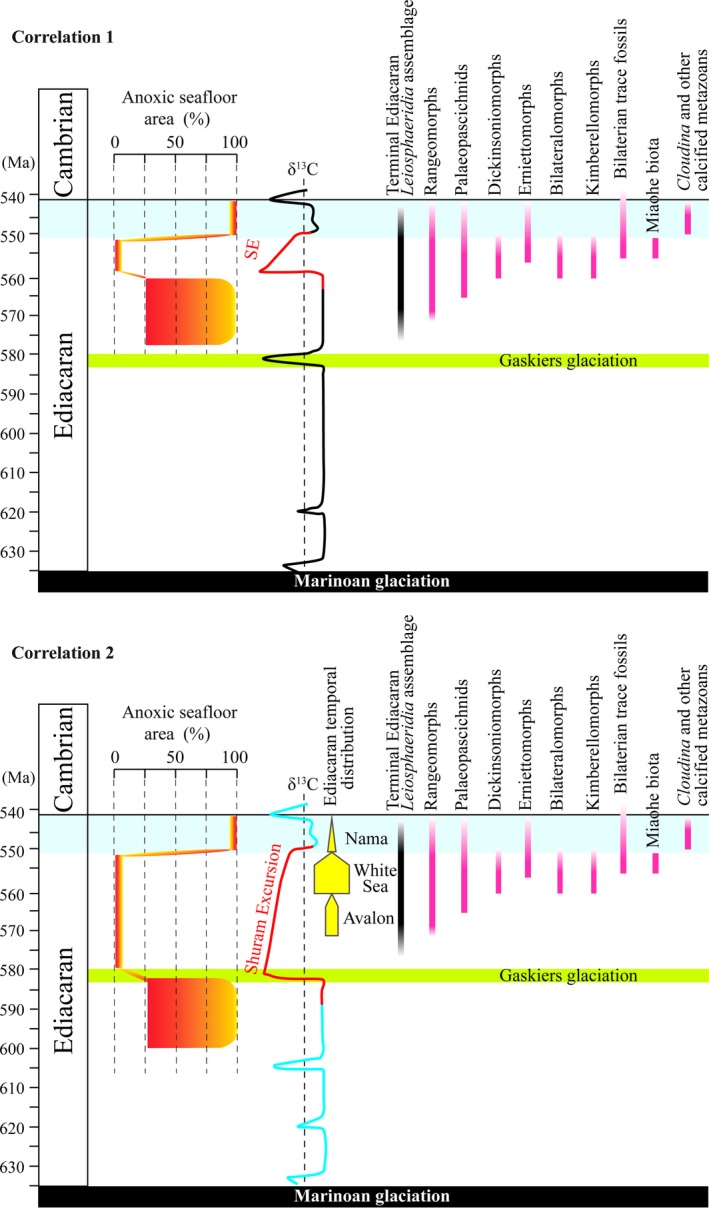
Correlation of marine redox evolution and the temporal distribution of macroscopic Ediacaran fossils. Correlations 1 and 2 are modified from Xiao et al. (2016). The anoxic seafloor area estimates shortly after the Shuram excursion (during the Doushantuo Member IV stage) and during the terminal Ediacaran Period (551–541 Ma) are based on δ^238^U data from Kendall et al. (2015) and from Zhang, Xiao et al. (2018), respectively [Colour figure can be viewed at http://wileyonlinelibrary.com]

The possible causal relationship between Ediacaran redox events and the evolution of the Ediacara biota is intriguing. The Ediacara biota contains three temporally successive assemblages that are reasonably constrained by radiometric dates (see summary in Xiao et al., 2016). These are the Avalon (~570–560 Ma ago), White Sea (~560–550 Ma ago), and Nama (~550–540 Ma ago) assemblages, which are named after representative geographic regions where they occur (Waggoner, 2003). However, as discussed above, the age and duration of the Shuram excursion are poorly constrained, and the temporal relationship between the SE and the Ediacara biota is uncertain. Given these uncertainties, we consider two end‐member scenarios (Figure 9): (a) The SE was initiated at ca. 560 Ma and lasted <10 Myr (i.e., correlation 1 of Xiao et al., 2016); and (b) the SE was initiated at ca. 580 Ma and lasted <30 Myr (i.e., correlation 2 of Xiao et al., 2016). If the SE started around 580 Ma (Witkosky & Wernicke, 2018), the new U isotope data presented here mean that the rise of the Ediacara biota, including the evolution of mobile animals as represented by putative trace fossils from the Avalon assemblage (Liu et al., 2010), closely followed (within <10 Myr) a global ocean oxygenation event around 580 Ma. On the other hand, if the Shuram started 560 Ma, it seems that the diversification of the Ediacara biota in the White Sea assemblage coincides with a global ocean oxygenation event around 560 Ma. The White Sea assemblage includes numerous taxa (e.g., kimberellomorphs, bilateralomorphs, and triradialomorphs) whose morphologies are consistent with higher minimum oxygen requirements compared with the majority of taxa from either the Avalon or Nama assemblages (Evans, Diamond, Droser, & Lyons, 2018). These taxa are prominently absent (and may have disappeared) from the Nama assemblage. Therefore, an episode of pervasive ocean oxygenation across the SE may have been an extrinsic driver either for the emergence of the Ediacara biota during the Avalon assemblage or its diversification in the White Sea assemblage. The subsequent shift to extensive anoxic conditions during the terminal Ediacaran Period coincides with the decline and extinction of the Ediacara biota (Tostevin et al., 2019; Wei et al., 2018; Zhang, Xiao, et al., 2018). Thus, although genetic, environmental, and ecological factors may have played a role in shaping the evolutionary history of early animals, our data suggest that the rise and fall of the Ediacara biota is, on the first order, coupled with the wax and wane of global ocean oxygenation.

## CONFLICT OF INTEREST

The authors declare no conflict of interest and no competing financial interests.

## AUTHOR CONTRIBUTIONS

F.Z. conceived the study, performed research and analyzed data; C.L., M.C., and S.W. contributed samples from the Jiulongwan section, sample details have been published in Li et al. (2017); D.H. contributed samples from the Death Valley section, sample details have been published in Hardisty et al. (2017); V.M., and B.P. contributed samples from the Siberia section, sample details have been published in Melezhik et al. (2005), Melezhik et al. (2009). All authors contributed to discussions. F.Z. wrote the manuscript with contributions from S.J.R., S.X., D.H., T.M.L., and A.D.A.
